# Genomic Instability in Peripheral Blood and Buccal Mucosal Cells of Marijuana Smokers: The Impact of Tobacco Smoke

**DOI:** 10.31557/APJCP.2020.21.5.1235

**Published:** 2020-05

**Authors:** Daniel Vitor De Souza, Samuel Rangel Claudio, Camila Lima Feitosa Da Silva, Kevin Pereira Marangoni, Rogerio Correa Peres, Daniel Araki Ribeiro

**Affiliations:** 1 *Department of Biosciences, Federal University of Sao Paulo, UNIFESP, Santos, SP, Brazil. *; 2 *University São Judas Tadeu, Campus UNIMONTE, Santos, SP, Brazil. *

**Keywords:** Oral mucosa cells, peripheral blood, marijuana, tobacco smoke, micronucleus test

## Abstract

**Background:**

The aim of this study was to evaluate cytotoxic, mutagenic and genotoxic effects on buccal mucosa and peripheral blood cells from marijuana and tobacco smokers.

**Methods:**

For this purpose, a total of 45 volunteers were distributed into four groups: CTRL group (control): individuals who did not smoke marijuana or tobacco (n = 11); Group M: Marijuana smokers (n = 13); Group T: Tobacco smokers (n = 13); Group M + T: Smokers of both marijuana and tobacco (n = 08).

**Results:**

Smokers of both marijuana and tobacco led an increase of micronucleated cells on buccal mucosa when compared to control group. The occurrence of karyolysis showed significant changes in this group as well. The comet assay data revealed genetic damage in peripheral blood cells for all groups of smokers.

**Conclusion:**

In summary, our results showed that marijuana and /or tobacco are able to induce genetic damage and cytotoxicity in oral and peripheral blood cells.

## Introduction

The plant Cannabis sativa belongs to the family Cannabaceae L, popularly known as marijuana from Asian continent (Lopez et al., 2014). The harvest occurs in the fall to have a good concentration of its main bioactive compound, Delta-9-tetrahydrocannabinol (THC) (Lopez et al., 2014). To date, there are several variables for consuming the plant, such as inhalation (smoking), ingestion (use of the plant in homemade recipes, such as cakes and biscuits) and skin absorption (adhesives). It is important to stress that the consumption method chosen directly affects the onset of its effect as well as the amount of THC that will be absorbed by the organism. The inhalation promoted by cigarettes in dry form is widely used due to greater effectiveness. It is estimated that 0.5-1 g of the plant contains 20 mg of THC. This in turn is consumed in the form of tetrahydrocannabinolic acid as a result of combustion of the plant (Bonfa et al., 2003). The acid then converts into free THC, where it is absorbed through inhalation; the smoke goes to the lungs allowing the bioactive substances to reach the bloodstream and central nervous system (Bonfa et al., 2003).

Marijuana is considered an illicit drug in many countries, such as Brazil, where it is widely consumed by people in general who justify its use to produce relaxation, and decreased stress and anxiety (Zuardi et al., 2010). Nevertheless, it has been documented that marijuana smoke promotes several harmful health effects (Lee and Hancox, 2011). In fact, some authors have postulated that marijuana smoke condensates contain similar chemical compounds as those detected in tobacco cigarette smoke (Moir et al., 2008). Many of them, have been classified as carcinogens by International Agency for Research on Cancer (IARC) (Smith et al., 2003). Herein, it would be important to know whether, and to what extent, marijuana smoke condensates, associated or not with tobacco cigarette smoke, could exert harmful effects on human health, especially on genetic material. This investigation is relevant since there are few studies investigating marijuana-only smoking populations as well as the association with tobacco cigarette smoke. 

To date, some research groups have investigated putative biomarkers for biomonitoring continuous exposure of human populations to environmental mutagens and carcinogens (Maranho et al., 2017; Claudio et al., 2019). Among them, micronucleus and single cell gel comet assays are simple, low cost, reproducible and non-invasive methodologies for investigating cytogenetic damage and DNA strand breaks in eukaryotic cells, respectively (Tice et al., 2000; Bonassi et al., 2011). Previous studies conducted by our research group have demonstrated that micronucleus and single cell gel comet assays are important tools for biomonitoring people exposed to chemical agents and/or conditions considered to be suspect (Guilheiro et al., 2014; Souza et al., 2016; Andrade et al., 2017; Da Silva et al., 2018).

The aim of this study was to investigate geno- and cytotoxicity in buccal mucosa and peripheral blood cells from marijuana users associated, or not, with cigarette smoke by micronucleus and single cell gel comet assays.

## Materials and Methods

*Participants*

For this study, a total of 45 volunteers were recruited. They were distributed into four groups, as follows: CTRL group (control): no smokers (n = 11); Group M: Marijuana smokers (n = 13); Group T: Tobacco smokers (n = 13); Group M + T: Smokers of both marijuana and tobacco (n = 08).The study’s eligibility criteria included: (iii) do not present chronic degenerative diseases, such as hypertension, diabetes or cancer, (iv) do not intake any medicines continuously; (v) do not present oral lesion when colleting oral cells; (vi) do not to be exposed to dental X-ray 1 month before collecting buccal cells; (vii) agree to participate in the study. Exposure to other known genotoxic agents, such as alcohol consumption was not recorded in this setting. The study was approved by the Ethics Committee of the University Sao Judas Tadeu, Campus UNIMONTE (Protocol number #2.222.726). Informed consent was obtained from all individuals included in the study. 

*Micronucleus test on oral mucosal cells*

Micronucleus test using buccal mucosa cells was made according to Belien et al., (1995). All slides were stained with Feulgen-Fast Green method being examined under a light microscope at ×1,000 magnification. Micronuclei were identified according to the criteria described by Belien et al., (1995) as a parameter of DNA damage (mutagenicity). For cytotoxicity, the following nuclear alterations were considered: pyknosis, karyolysis and karyorrhexis (Tolbert et al., 1992). This analysis was performed by one experienced observer. A total of 2000 cells were evaluated per volunteer.

*Single cell gel comet assay in peripheral blood cells*

The single cell gel comet assay was performed to peripheral blood cells as described by Tice et al., (2000). A total of 25 comets were evaluated per volunteer. To measure the genotoxicity, tail moment was chosen. This is defined as the product between % comet tail and DNA migration. The value was expressed to arbitrary units. 

*Statistical methods*

The Kruskall-Wallis non-parametric test followed by Dunn’s test were used to compare the frequencies of cytotoxicity among the samples between the experimental group versus control group. Micronucleus frequencies among groups were evaluated as established by Pereira (1991). The tail moment data was evaluated by two-way ANOVA (analysis of variance) followed by Tukey’s test. The statistical analysis was conducted using BioStat software version 5.0 (Maringa. PR. Brazil). The level of significance was set at 5%.

## Results

The demographic characteristic for all participants are presented in Table 1.

The results of this study demonstrated that marijuana did not increase the total number of micronucleated cells. Cytotoxicity did not show statistically significant differences (p>0.05). Such findings are demonstrated in Table 2.

As it was evidenced with marijuana smokers, Tobacco cigarette smokers did not show any significant differences with respect to the number of micronucleated cells when compared to control group. Regarding metanuclear changes indicative of cytotoxicity, the same picture occurred, i.e. no statistically significant differences (p>0.05) were found to pyknosis, karyorrhexis and karyolysis. 

**Figure 1:**
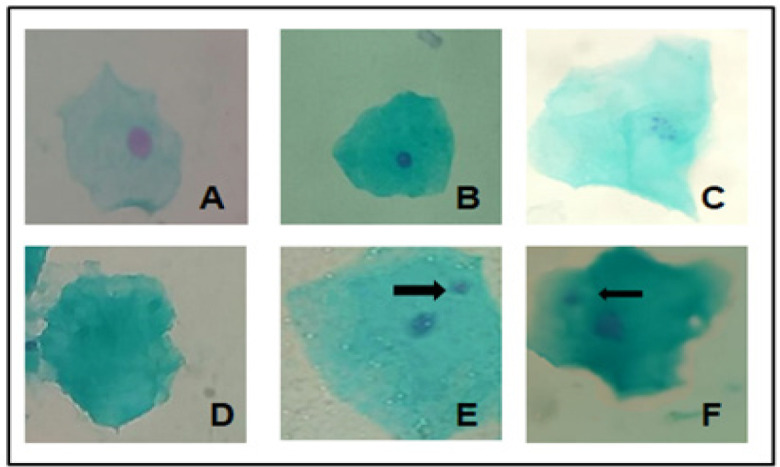
Photomicrography of Oral Mucosa Cells Cells Showing Metanuclear Changes. (A), Normal cell; (B), Pyknosis; (C), Karyorrhexis; (D), Karyolysis; E and F, Micronucleus (arrow). Feulgen-Fast green staining. X100 magnification

Interestingly, smokers of both marijuana and Tobacco demonstrated an increase of micronucleated cells when compared to control group (p<0.05). For cytotoxicity, the occurrence of karyolysis showed significant changes (p<0.05) as well. With respect to pyknosis and karyorrhexis, no remarkable changes (p>0.05) were detected in this group. Such findings are shown in Table 2. Figure 1 illustrates all metanuclear changes considered in this study (pyknosis, karyorrhexis, karyolysis and micronucleus).

**Figure 2:**
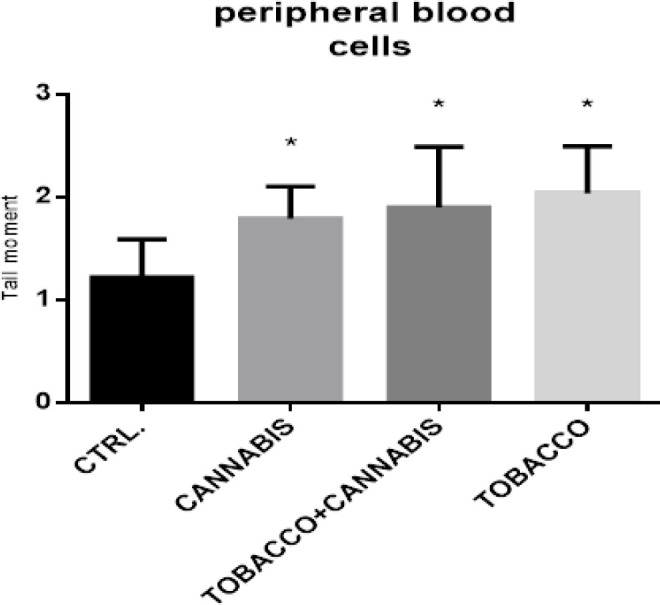
Single Cell Gel Comet Assay Data (Tail Moment) in Peripheral Blood Cells from Marijuana and/or Tobacco Users. Results are expressed as Mean ± S.D. P<0.05 when compared to control group

Finally, the single cell gel comet assay data revealed that all experimental groups showed DNA damage in peripheral blood cells. Statistically significant differences (p<0.05) were noticed with marijuana and/or Tobacco smokers when compared to control group. These findings are demonstrated in Figure 2. 

## Discussion

The main results of this study revealed that smokers of both marijuana and tobacco led an increase of micronucleated cells on buccal mucosa as well as DNA breakage in peripheral blood cells. There are few studies regarding the consumption of marijuana smoke condensates since the majority of investigations are conducted with isolated compounds, such as Δ9-THC or cannabidiol. 

First, our results revealed that marijuana was able to induce genetic damage in peripheral blood cells. However, it not possible to identify an increase in the frequency of micronucleated cells on buccal mucosa in marijuana smokers. In a similar way, no significant differences with respect to cytotoxicity were detected. Having searched the scientific literature, it was noted that the genotoxicity of marijuana has been well documented. DNA damage in macrophages from the lungs of marijuana smokers was found by some authors (Sherman et al. 1995). Marijuana smoke condensates were mutagenic in the Ames test as well (Maertens et al., 2009). Some components induced cytogenetic damage in mammalian cells in vivo and in vitro (Maertens et al., 2013; Lucić Vrdoljak et al., 2019; Russo et al., 2019). Moreover, non-mammalian species demonstrated positive genotoxicity induced by marijuana (Parolini et al., 2017). An epidemiological study showed that the continuous use of cannabis has a mutagenic effect (Zhang et al., 1999). Following the rationale, several biological mechanisms have been proposed for elucidating the genotoxicity induced by marijuana, such as the generation of reactive oxygen species (ROS) and deregulation of apoptosis by means of abnormal p53 expression (Kim et al., 2013). It has been demonstrated that a brief exposure to Cannabis sativa induced the formation of ROS associated with significant reduction of GSH levels, leading to oxidative stress in the cell (Sarafian et al., 1999). Probably, the quantity of tobacco cigarettes smoked by people was not enough to demonstrate an in vivo effect in this setting. Further studies are necessary to clarify the issue.

The results obtained from the tobacco cigarette smokers failed to detect a positive effect in the micronucleus assay. Regarding cytotoxicity, no significant changes were detected by means of pyknosis, karyolysis and karyorrhexis in this study. However, tobacco cigarette smokers presented genetic damage in peripheral blood cells. Tobacco smoke is able to deregulate intrinsic apoptosis pathways, and to modulate the expression of some xenobiotics metabolizing proteins (Assis et al., 2005; Ribeiro and Assis, 2008). Furthermore, the biological relevance of tobacco cigarette smoke, when studying the micronucleus assay on oral cells, is well established in scientific literature. It seems that tobacco cigarette smoke induces metanuclear changes in buccal mucosa cells (Nersesyan et al., 2006; Pereira da Silva et al., 2015; Metgud and Neelesh, 2018). Nevertheless, Nersesyan et al., (2011) have postulated that the occurrence of such nuclear alterations is dependent on type and quantity of tobacco cigarettes smoked per day. Therefore, it is assumed that the number of cigarettes consumed is mandatory for detecting any biological effect. Certainly, this explains our results since the participants smoke only a few cigarettes per day. 

Considering that a large number of marijuana smokers are also tobacco cigarette smokers, we decided to investigate the combination of the two. Our results revealed the presence of DNA strand breaks in peripheral blood cells and increased number of micronucleated cells in this group. An increase in the frequency of karyolysis was also found in these volunteers as well. Some authors have revealed analogous compounds between marijuana and tobacco smoke products (Maertens et al., 2009). Such information helps to explain that the association plays an important role for genotoxic, mutagenic and cytotoxic properties exerted by marijuana on oral and peripheral blood cells. In fact, some authors pointed out the positive correlation between cannabis and cancer. Hirao-Suzuki (2019) showed that Δ9-THC increases fatty acid 2-hydroxylase (*FA2H*) expression in human breast cancer cells. Murphy et al., (2019) have assumed that cannabis use was associated with significantly lower sperm concentration and Callaghan et al., (2011) recently found a link between cannabis consumption and testicular cancer. However, some studies have postulated that cannabis inhibited tumor development. Shrivastava et al., (2011) have demonstrated that cannabidiol is able to kill breast cancer cells by inducing oxidative stress and inhibiting mTOR signaling. One possible explanation for these controversial results may be the form of administration of cannabis. When smoked, cannabis seems to lose its protective effects as far as to induce harmful effects. When both are smoked, our results demonstrate that marijuana smoke condensates, when associated with cigarette smoke, induce genetic damage and cellular death in peripheral blood and buccal mucosal cells. 

In summary, our results showed that a combination of marijuana and tobacco smoking habits is able to induce genomic instability in peripheral blood and buccal mucosa cells. Certainly, this comprises a risk condition for chemical carcinogenesis. Even so, it is important to stress the difficulty in designing the study. This is because the consumption of marijuana is illegal here in Brazil. Therefore, recruiting volunteers to join and participate was quite complicated in this study. For this reason, the number of volunteers was relatively small. Further analysis with a larger sample of volunteers continuously exposed to cannabis is interesting as a future perspective to confirm the data obtained.

**Table 1:** Demographic Characteristics from All Participants of the Study

Groups	CTRL(n=11)	M (n=13)	T (n=13)	M+T (n=08)
Age (years)	21+8.1	25+6.9	22+3.0	26+8.7
Gender	M / F	M / F	M / F	M / F
	4/7	9/4	5/8	1/7
Average user time of Marijuana (years)	-	6.76	-	11
Average user time of Tobacco (years)	-	-	7.92	9
Frequency of use of Marijuana	-	From 1 to 7 days a week	-	7 days a week
Frequency of use of Tobacco	-	-	From 2 to 7 days per week	7 days per week

CTRL, control group; M, marijuana group; T, tobacco group; M+T, marijuana and tobacco group.

**Table 2:** Mean and S.D of Pyknosis, Karrhyorexis, Karyolysis and Micronucleus on Buccal Mucosa Cells of Marijuana and/or Tobacco Users

Groups	Pyknosis	Karrhyorexis	Karyolysis	Micronucleus
CTRL	272.5 ± 193.5	5.54 ± 8.21	430.2 ± 522.5	0.10 ± 0.15
M	177.9 ± 216.3	7.15 ± 9.90	618.9 ± 512.6	0.15 ± 0.37
T	56.54 ± 79.06	4.53 ± 3.88	350.8 ± 317.3	0.23 ± 0.59
M+T	114.5 ± 137.3	2.62 ± 3.62	1045 ± 417.6*	0.50 ± 1.06*

CTRL, control group; M, marijuana group; T, tobacco group; M+T, marijuana and tobacco group; *p<0.05 when compared to control group
